# Circ‐IGF1R inhibits cell invasion and migration in non‐small cell lung cancer

**DOI:** 10.1111/1759-7714.13329

**Published:** 2020-02-27

**Authors:** Zhanyu Xu, Weiwei Xiang, Wenjie Chen, Yu Sun, Fanglu Qin, Jiangbo Wei, Liqiang Yuan, Liping Zheng, Shikang Li

**Affiliations:** ^1^ Department of Thoracic and Cardiovascular Surgery The First Affiliated Hospital of Guangxi Medical University Nanning China; ^2^ Department of Anesthesia Catheter Room The First Affiliated Hospital of Guangxi Medical University Nanning China

**Keywords:** Circ‐IGF1R, circular RNA, invasion, migration, NSCLC

## Abstract

**Background:**

Circular RNA (circRNA) is a novel molecular marker and target candidate that is closely associated with tumor invasion and migration. The mechanism of action of hsa_circ_0005035 (circ‐IGF1R) in non‐small cell lung cancer remains unclear. In this study, we aimed to study the mechanism of action of circ‐IGF1R in lung cancer.

**Methods:**

We screened circ‐IGF1R, one of the most notable differential expressions, from the Gene Expression Omnibus database, GSE104854, for further research. The expression level of circ‐IGF1R was examined using quantitative reverse transcription‐polymerase chain reaction (qRT‐PCR) in five different lung cancer cell lines and 50 pairs of lung cancer and adjacent tissues. Wound‐healing and Transwell assays were used for verifying the biological function of circ‐IGF1R. The effect of overexpressing circ‐IGF1R on the transcriptome of whole lung cancer cells was explored in lung cancer cell lines using RNA‐seq.

**Results:**

The expression level of circ‐IGF1R was notably lower in lung cancer tissues and lung cancer cell lines than in the adjacent normal tissues and cells (*P* < 0.0001). In addition, the expression level of circ‐IGF1R was associated with larger tumors (T2/T3/T4) and lymph node metastasis (N1/ N2/N3) (*P* < 0.05). The overexpression of circ‐IGF1R significantly inhibited the invasion and migration of the lung cancer cells. The potential network of circ‐IGF1R–miR‐1270–VANGL2 was preliminarily determined, and the expression patterns of miR‐1270 and VANGL2 were verified in lung cancer cell lines.

**Conclusion:**

Circ‐IGF1R may inhibit lung cancer invasion and migration through a potential network of circ‐IGF1R–miR‐1270–VANGL2.

## Introduction

Lung cancer is a malignant tumor that occurs worldwide. According to statistics presented by the World Health Organization (WHO), lung cancer has the highest incidence (11.6% of new cancers worldwide) and mortality rate (18.4% of global cancer deaths) among new global cancer cases in 2018.^1^ Non‐small cell lung cancer (NSCLC) is distinguished from small cell lung cancer (SCLC) by its clinical symptoms and degree of progression, and it accounts for the vast majority (85%) of lung cancers. The five‐year survival rate for patients with early‐stage NSCLC after surgical treatment is 70%–90%, and approximately three‐quarters of patients lose their chances of surgery at the time of the initial diagnosis.^2^ In recent years, important breakthroughs in targeted molecular therapy and immunotherapy for lung cancer have demonstrated an urgent requirement of identifying novel biomarkers for different populations. This will have important implications for improving patients' early diagnosis rates and for discovering novel targeted therapies.

Circular RNA (circRNA) is a circularly closed RNA that is formed by covalent bonding (as opposed to a linear RNA lacking free ends). This circular structure makes circRNA resistant to exonuclease activity. Thus, circRNA exhibits excellent stability in its intracellular half‐life, which is more than 48 hours.^3^ In addition, circRNA is abundantly expressed in eukaryotes, and its expression is tissue‐specific and disease‐specific^4^; it can thus be investigated whether circRNA plays a regulatory role in tissue growth and development and as a diagnostic marker for specific tissue diseases.

CircRNA primarily affects gene expression at the transcriptional or post‐transcriptional level. The biological functions of circRNA include the following: functionality as an *miRNA* sponge, the regulation of selective splicing and gene transcription, interaction with RNA‐binding proteins, and protein translation.^5^, ^6^, ^7^, ^8^ Studies have shown a close relationship between circRNA and a variety of tumors. Cir‐ITCH can adhere to miR‐7 and miR‐214, thereby aggravating the inhibitory effect of ITCH on the downstream Wnt pathway.^9^ Hsa_circRNA_103809 adheres to miR‐4302, thereby increasing the proliferation and migration of lung cancer cells produced by MYC.^10^ Hsa_circ_0013958 was identified as a sponge for miR‐134, which promotes the progression of lung cancer by upregulating cyclin D1.^11^ At present, the role of circ‐IGF1R in lung cancer is still unclear, and its specific mechanisms are yet to be elucidated.

In the present study, we first assessed the expression and biological function of circ‐IGF1R in NSCLC tissues and cells, and we performed RNA‐seq in lung cancer cell lines overexpressing circ‐IGF1R to further explore its mechanism of action.

## Methods

### Bioinformatics analysis

The high‐throughput RNA sequencing data of the circRNA profile, GSE104854, of NSCLC was retrieved from the Gene Expression Omnibus database (GEO, http://www.ncbi.nlm.nih.gov/geo). The circ‐IGF1R miRNA target gene prediction was conducted using https://circinteractome.nia.nih.gov/website.

### Cell lines

NSCLC cell lines (PC9, A549, Calu‐1, H1299, and H1975) and MRC‐5 (a normal human embryonic lung fibroblast cell line) were purchased from the Type Culture Collection of the Chinese Academy of Sciences (Shanghai, China) and incubated in RPMI‐1640 medium (Gibco, NY, USA) containing 10% fetal bovine serum (100 ug/mL streptomycin, 100 U/mL penicillin, and 1.5 mg/L glutamine), at 37°C in a 5% CO_2_‐saturated humidified incubator.

### Cell transfection

Small interfering RNA (siRNA) of circ‐IGF1R used to transfect A549 and PC9 cell lines was provided by GenePharma (Shanghai, China), and the sequences were as follows: si‐circ‐IGF1R sense, 5′‐GAAAATCTGCGGGCCAGGCAT‐3′; and antisense, and 5′‐ATGCCTGGCCCGCAGATTTTC‐3′. The circ‐IGF1R overexpression plasmid was purchased from General Biosystems (Hefei, China) and transfected into A549 and PC9 cell lines to overexpress circ‐IGF1R (ov‐circ‐IGF1R). Lipofectamine 2000 (Invitrogen, Carlsbad, CA) was utilized for all transfections in accordance with the manufacturer's instructions.

### Sample collection

A total of 50 NSCLC tissues and matched adjacent noncancerous lung tissue samples were collected from the First Affiliated Hospital of Guangxi Medical University between February 2015 and September 2018. The diagnosis of NSCLC was confirmed by pathologists. All samples were collected with written consent from patients. The ethics committee of the First Affiliated Hospital of Guangxi Medical University approved this study. All tissue specimens were preserved at −80°C prior to further analysis.

### RNA isolation and qRT‐PCR

In accordance with the manufacturer's instructions, the TRIzol kit (Invitrogen, Carlsbad, CA, USA) was used to isolate total RNA from lung tissues and cell lines. Primers were designed and synthesized by Cellcook (Guangzhou, China). The qRT‐PCR analysis was performed on the ABI7300 system (Applied Biosystems) using the SYBR green kit (TaKaRa, Dalian, China) as per the manufacturer's protocol. Glyceraldehyde 3‐phosphate dehydrogenase (GAPDH) or U6 served as the endogenous control. The primers were utilized as shown in Table 1. The relative quantification values were determined using the comparative Ct method (2^−ΔΔCt^).

**Table 1:** Primer sequences used in this study

Name	Forward Primer (5′–3′)	Reverse Primer (5′–3′)
Circ‐IGF1R_divergent	CAAACCGCTGCCAGAAAATCT	ACTCGGTAATGACCGTGAGC
Circ‐IGF1R_convergent	AGGCTGGGGCTCTTGTTTAC	GCGTCCTCTCGCTCTC
GADPH divergent	TCCTCACAGTTGCCATGTAGACCC	TGCGGGCTCAATTTATAGAAACCGGG
GADPH convergent	GAGTCAACGGATTTGGTCGT	GACAAGCTTCCCGTTCTCAG
Hsa‐miR‐1270	CTGGAGATATGGAAGAGCT	GTCGTATCCAGTGCAGGGTCCGAGGTATTCGCACTGGATACGACACACAG
Hsa‐miR‐432‐5p	TCTTGGAGTAGGTCATTGG	GTCGTATCCAGTGCAGGGTCCGAGGTATTCGCACTGGATACGACCCACCC
Hsa‐miR‐377‐3p	ATCACACAAAGGCAACTT	GTCGTATCCAGTGCAGGGTCCGAGGTATTCGCACTGGATACGACACAAAA
Hsa‐miR‐194‐3p	CCAGTGGGGCTGCTGTTA	GTCGTATCCAGTGCAGGGTCCGAGGTATTCGCACTGGATACGACCAGATA
Hsa‐miR‐1299‐3p	CTCTCACCACTGCCCTCCC	GTCGTATCCAGTGCAGGGTCCGAGGTATTCGCACTGGATACGACCTGTGG
Hsa‐miR‐653‐5p	GTGTTGAAACAATCTCT	GTCGTATCCAGTGCAGGGTCCGAGGTATTCGCACTGGATACGACCAGTAG
Hsa‐miR‐515‐5p	TTCTCCAAAAGAAAGCACTT	GTCGTATCCAGTGCAGGGTCCGAGGTATTCGCACTGGATACGACCAGAAA
Hsa‐miR‐766	ACTCCAGCCCCACAGCCT	GTCGTATCCAGTGCAGGGTCCGAGGTATTCGCACTGGATACGACGCTGAG
Hsa‐miR‐1294	TGTGAGGTTGGCATTGTT	GTCGTATCCAGTGCAGGGTCCGAGGTATTCGCACTGGATACGACAGACAA
VANGL2	GGCTCCCGATCTGATTCCTG	TCTTGAGTCTGGTCACCCCC
VIM	AGTCCACTGAGTACCGGAGAC	CATTTCACGCATCTGGCGTTC
CTNNB1	AACTTGCCACACGTGCAATC	AGGTTATGCAAGGTCCCAGC

### Western blot

In lung cancer cells A549 and PC9 that overexpress circ‐IGF1R and interfere with circ‐IGF1R, the total protein was extracted using RIPA lysis buffer, respectively. SDS‐PAGE was conducted to separate proteins, which were blotted onto PVDF membrane. The primary antibodies anti‐VANGL2 (ab198887, Abcam, UK) and anti‐GAPDH (60004‐1, Proteintech, USA) were diluted 1:2000 and 1:5000 according to the instructions and incubated overnight at 4°C. HRP rabbit IgG secondary antibodies were added at a dilution of 1:2000 and incubated for one hour at room temperature. Signals were detected using ECL detection reagent (Millipore) following the manufacturer's instructions.

### Cell invasion and migration assay

For cell invasion, the Corning Transwell chambers were used. Briefly, PC9 and A549 cells were fostered in 24‐well plates. After incubation for 48 hours, the cells on the lower surfaces were fixed in 4% formaldehyde, stained with 0.25% crystal violet, and counted. A wound‐healing assay was performed to evaluate cell migration. PC9 cells and A549 cells were fostered in six‐well plates (Corning) and grown to 80%–90% confluence. Wounds were then prepared using a yellow pipette tip and photographed at 0, 24, and 48 hours.

### RNA‐seq and differential expression analysis

For ov‐NC and ov‐circ‐IGF1R PC9 and A549 cell lines, following RNA isolation, the quantity of total RNA from samples was measured using the Qubit RNA Assay Kit (Life Technologies, Inc.) and the quality of RNA was assessed using the Agilent 2100 Bioanalyzer and RNA6000 Nano Kit (Agilent Technologies, Inc). The sequencing library was prepared as per the manufacturer's instructions for the NEBNext Ultra™ RNA Library prep kit for the Illumina platform. Next, we analyzed the functions of differentially expressed genes (DEGs). The overexpression of DEGs and negative control samples in the A459 and PC9 cells were selected using log2Ratio ≥ 1 (OV_circcirc‐IGF1R/OV_NC), as well as the false discovery rate (FDR) of multiple hypothesis testing (*P* < 0.001). Thus, a functional enrichment analysis of the Gene Ontology (GO) and the Kyoto Encyclopedia of Genes and Genomes (KEGG) were consulted to infer circ‐IGF1R function. Further, we combined the analysis results from Circinteractome (https://circinteractome.nia.nih.gov/index.html) with our RNA‐seq data. Circ‐IGF1R was utilized as a seed to enrich the circRNA–miRNA–mRNA interaction network established using Cytoscape software.

### Statistical analysis

Student's *t*‐test was used to compare the two groups. All statistical analyses were performed using SPSS 22.0 and GraphPad Prism 8.0 software. *P* < 0.05 was considered statistically significant.

## Results

### Investigation of differentially expressed circRNAs in lung carcinoma and verification of expression level using qRT‐PCR

**Figure 1:**
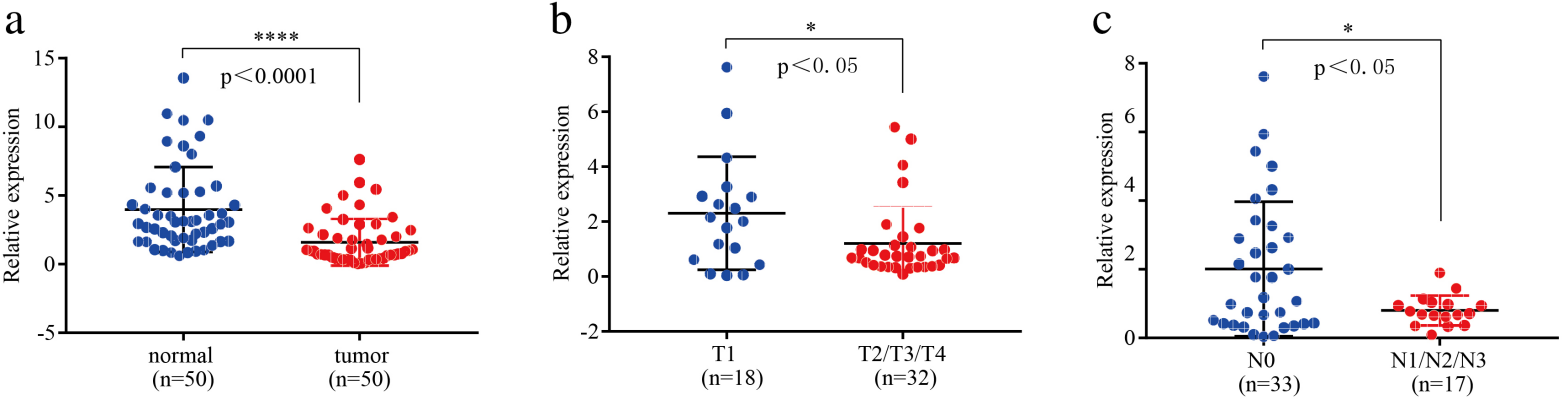
Circ‐IGF1R expression was downregulated in NSCLC cells and tissues. (**a**) The expression level of circ‐IGF1R in 50 NSCLC cancer tissues and adjacent normal tissues. (**b**) The expression level of circ‐IGF1R in lung cancer tissues of patients in the T1 and the T2–4 groups. (**c**) The expression level of circ‐IGF1R in lung cancer tissues of patients in the N0 and the N1–3 groups.

To investigate the differential expression of circRNA in lung cancer tissues, we analyzed high‐throughput sequencing data for three pairs of lung cancer tissues (lung adenocarcinoma and corresponding adjacent tissues) in GSE104854 from the GEO database. The results identified 56 circRNAs in lung cancer tissues compared with adjacent tissues. Among the 56 circRNAs, 44 were upregulated and 12 were downregulated. To verify the expression levels of the four most prominent circRNAs in lung cancer tissues, we selected 50 pairs of NSCLC tissues and adjacent tissues for qRT‐PCR. The results showed that the expression level of circ‐IGF1R in NSCLC tissues was significantly lower than that in the noncancerous tissues (*P* < 0.0001) (Fig 1a). Therefore, we selected circ‐IGF1R for further research.

### Relationship between expression level of circ‐IGF1R and clinical characteristics

With regard to whether circ‐IGF1R is associated with the clinical characteristics of lung cancer, the analysis showed that the abnormal expression of circ‐IGF1R was associated with the T stage (*P* = 0.0272) (Fig 1b) and the N stage (*P* = 0.0159) (Fig 1c). The T stage indicated the tumor size and invasion range, while the N stage indicated whether the tumor was associated with lymph node metastasis. The expression of circ‐IGF1R in the present study correlated with tumor and lymph node metastasis. However, no marked correlation was observed between circ‐IGF1R expression and other clinical pathological factors, including sex, age, smoking, subtype, and pathologic TNM (Tumor, Node, Metastasis) stage (Table 2).

**Table 2:** Circ‐IGF1R expression and clinicopathological features in patients with NSCLC

Variable	Patients	Mean ± SD	*P*‐value
Age, years			0.451
≤60	25	1.899 ± 0.4531	
>60	25	1.483 ± 0.3064	
Sex			0.2049
Male	31	1.419 ± 0.2997	
Female	19	2.135 ± 0.5189	
Smoking history			0.2904
Yes	30	1.928 ± 0.4083	
No	20	1.335 ± 0.292	
T			0.0272*
T1	18	2.303 ± 0.485	
T2/T3/T4	32	1.204 ± 0.2391	
N			0.0159*
N0	33	2.011 ± 0.3414	
N1/N2/N3	17	0.8007 ± 0.1061	
Subtype			0.7061
Lung adenocarcinoma	40	1.743 ± 0.3188	
Squamous cell carcinoma	10	1.483 ± 0.5004	
TNM stage			0.3425
I–II	37	1.846 ± 0.3432	
III–IV	13	1.25 ± 0.3702	

### Detection of circ‐IGF1R expression in lung cancer cells

**Figure 2:**
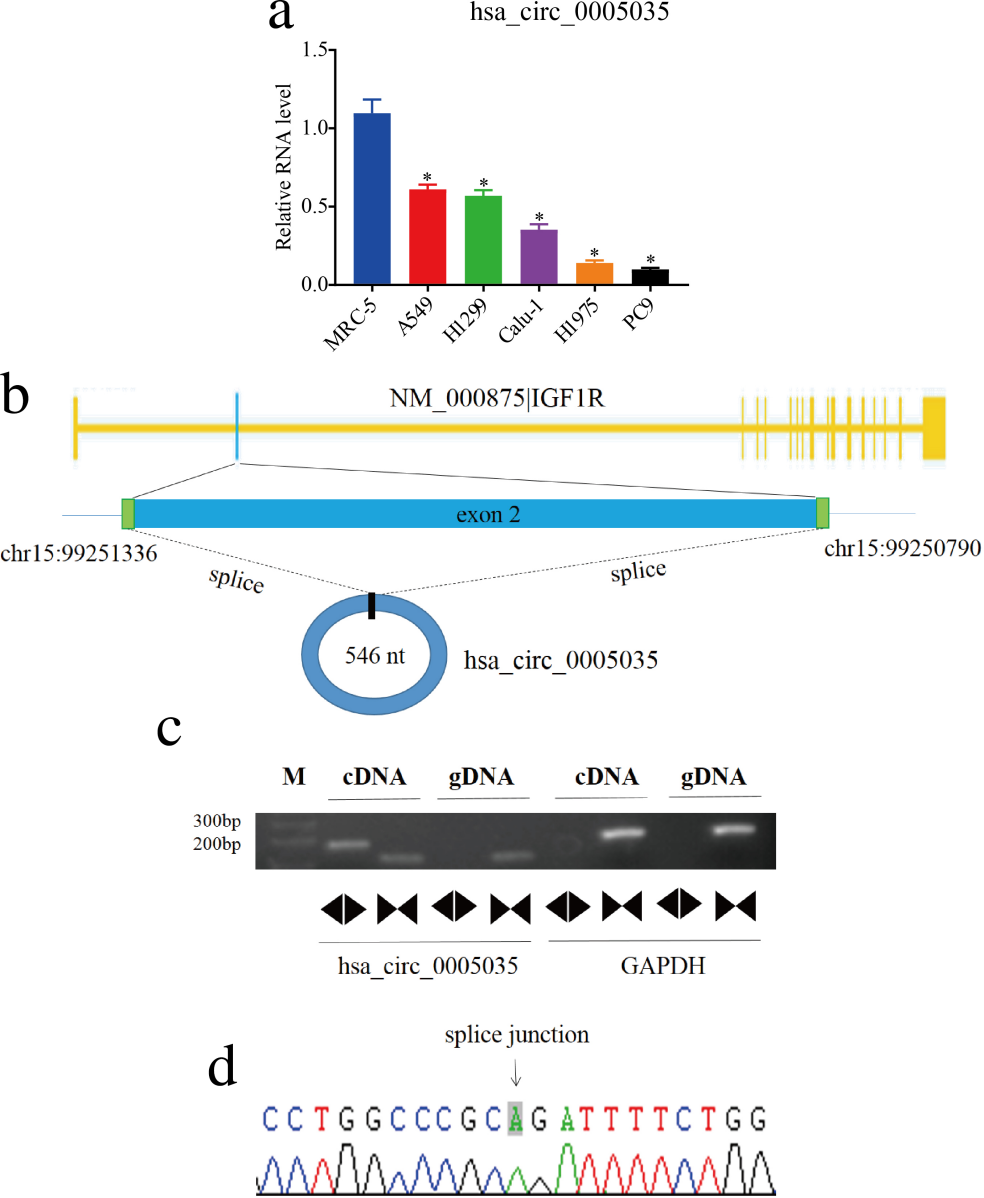
Detection of circ‐IGF1R in lung cancer cells. (**a**) The relative expression levels of circ‐IGF1R in six cell lines. (**b**) Schematic demonstrating that circ‐IGF1R is produced at the IGF1R gene locus exon2. (**c**) qRT‐PCR assay with divergent or convergent primers affirming the existence of circ‐IGF1R in A549 cells. GAPDH was used as the negative control. (**d**) Sanger sequencing of circ‐IGF1R verified the back‐splice junction.

To identify the expression of circ‐IGF1R in lung cancer cells, we first screened for circ‐IGF1R in six cell lines using qRT‐PCR. The results showed that the expression of circ‐IGF1R in lung cancer cells (PC9, A549, Calu‐1, H1299, and H1975) was significantly lower than that in the control cell line, MRC‐5 (Fig 2a). In addition, circ‐IGF1R is located on chromosome 15q26.3 and consists of exon2 (Fig 2b). Reverse splicing of circ‐IGF1R was successfully confirmed using Sanger sequencing (Fig 2c,d).

### Biological function of circ‐IGF1R in lung cancer cells

**Figure 3:**
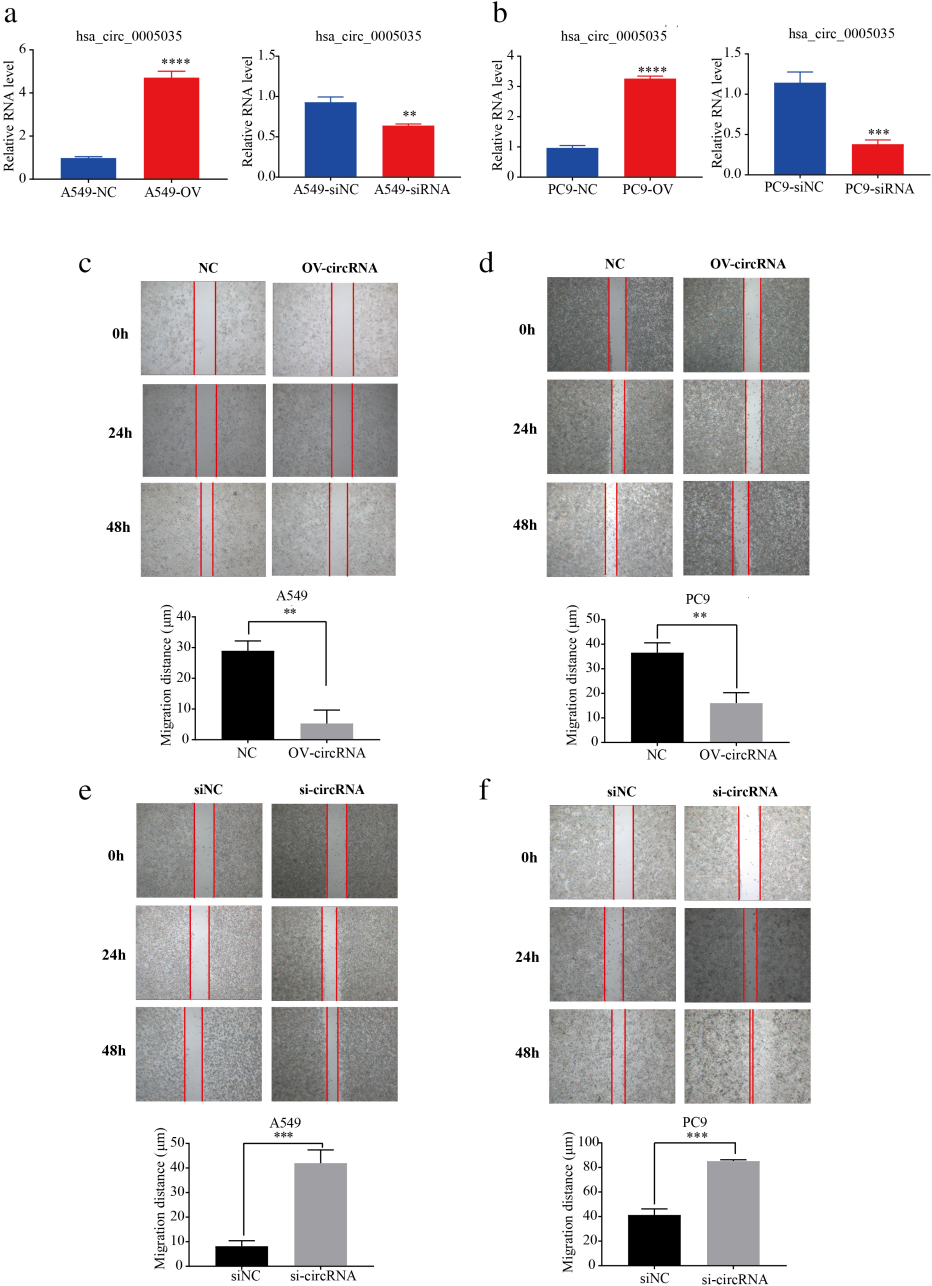
Circ‐IGF1R inhibited migration of lung cancer A549 and PC9 cell lines. The mRNA expression of circ‐IGF1R was detected using qRT‐PCR after overexpression or interference with circ‐IGF1R in lung cancer A549 (**a**) and PC9 (**b**). A wound‐healing assay was performed in A549 (**c**) and PC9 cells (**d**) after overexpressing circ‐IGF1R. (*P* = 0.0017 vs. NC; and *P* = 0.0037 vs. NC). Cell migration was detected using a wound‐healing assay after interference with circ‐IGF1R in A549 (**e**) and PC9 (**f**) cell lines (*P* = 0.0006 vs. siNC; and *P* = 0.0001 vs. siNC).

**Figure 4:**
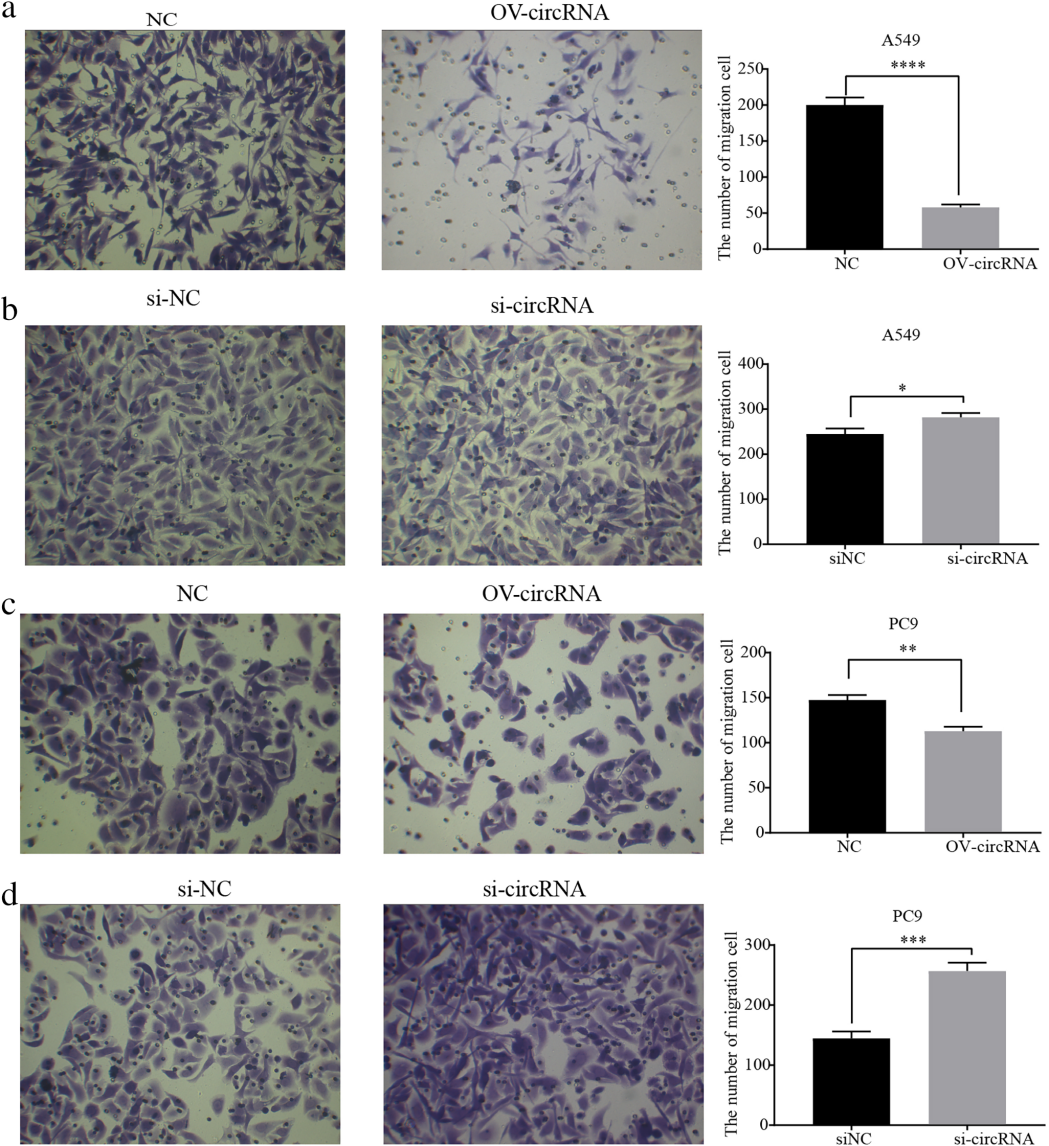
Circ‐IGF1R inhibited invasion of lung cancer A549 and PC9 cell lines. Invasion of cells after overexpression of circ‐IGF1R by Transwell assay in lung cancer A549 (**a**) and PC9 (**c**) cell lines was significantly inhibited (*P* < 0.0001 vs. NC; and *P* = 0.0013 vs. NC). The invasive ability of circ‐IGF1R was significantly increased by Transwell assay in lung cancer A549 (**b**) and PC9 (**d**) cell lines (*P* = 0.0151 vs. siNC; and *P* = 0.0004 vs. siNC).

The research team successfully constructed a plasmid that overexpressed the following: (i) circ‐IGF1R; and (ii) siRNA against circ‐IGF1R. These plasmids were transfected into human NSCLC A549 and PC9 cell lines. The wound‐healing and Transwell assays were used to verify the different functional effects of cancer cells. The overexpression of circ‐IGF1R significantly inhibited invasion and migration of lung cancer cells, and the opposite effect was observed when siRNA against circ‐IGF1R was used (Figs 3 and 4).

### CircIGF1R may affect the Wnt signaling pathway–related target gene, *VANGL2*

**Figure 5:**
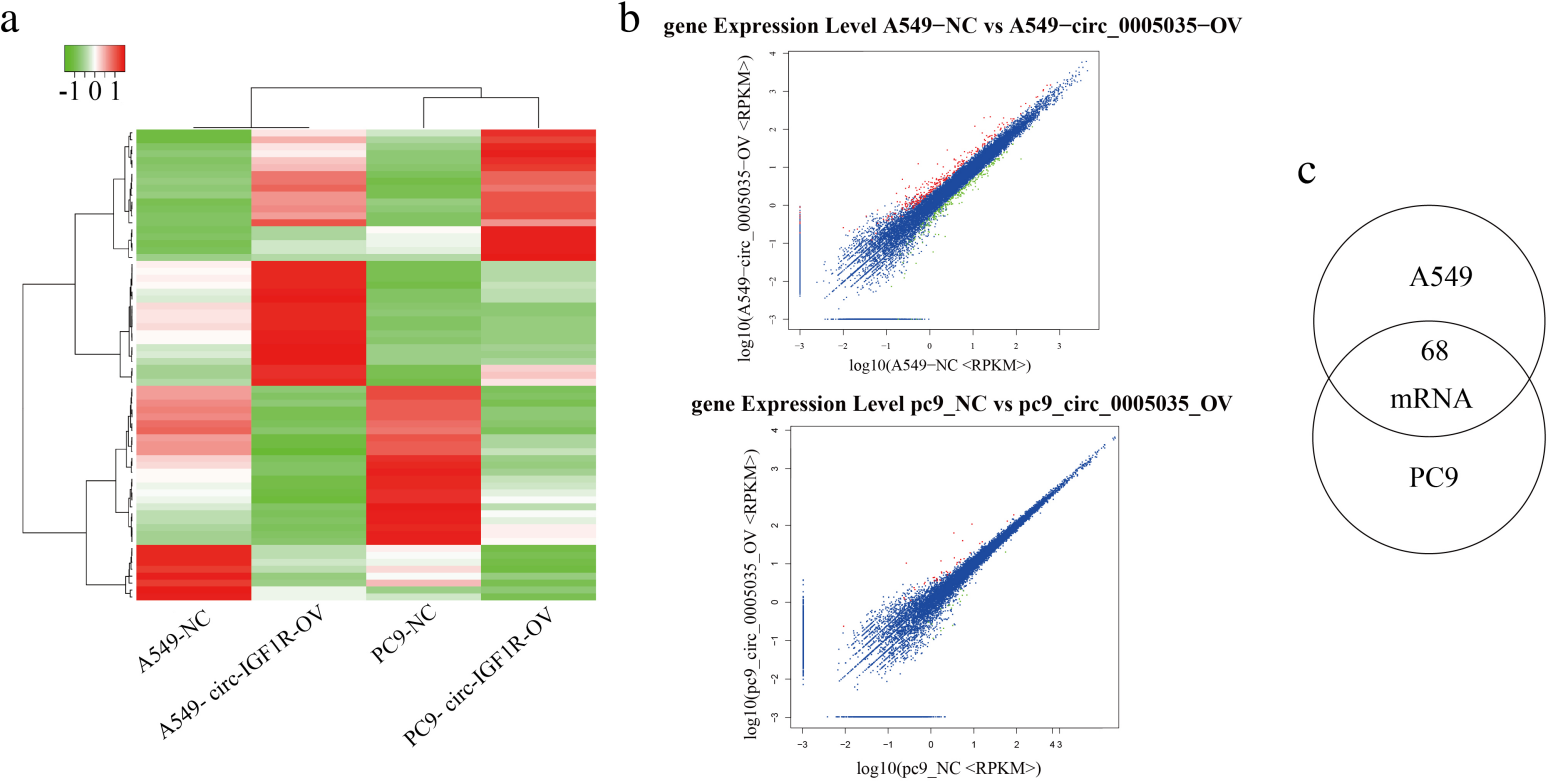
mRNAs regulated by circ‐IGF1R. (**a**) The cluster heat map showing the differentially expressed mRNAs. (**b**) A scatter plot was used to evaluate the variation in mRNA expression between ov‐NC and ov‐circ‐IGF1R of PC9 (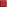) upregulated tags, (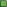) downregulated tags, and (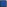) Not DETs and A549 cells (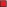) upregulated tags, (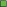) downregulated tags, and (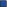) Not DETs. (**c**) Venn diagrams show common differentially expressed mRNAs between ov‐NC and ov‐circ‐IGF1R in PC9 and A549 cells.

**Figure 6:**
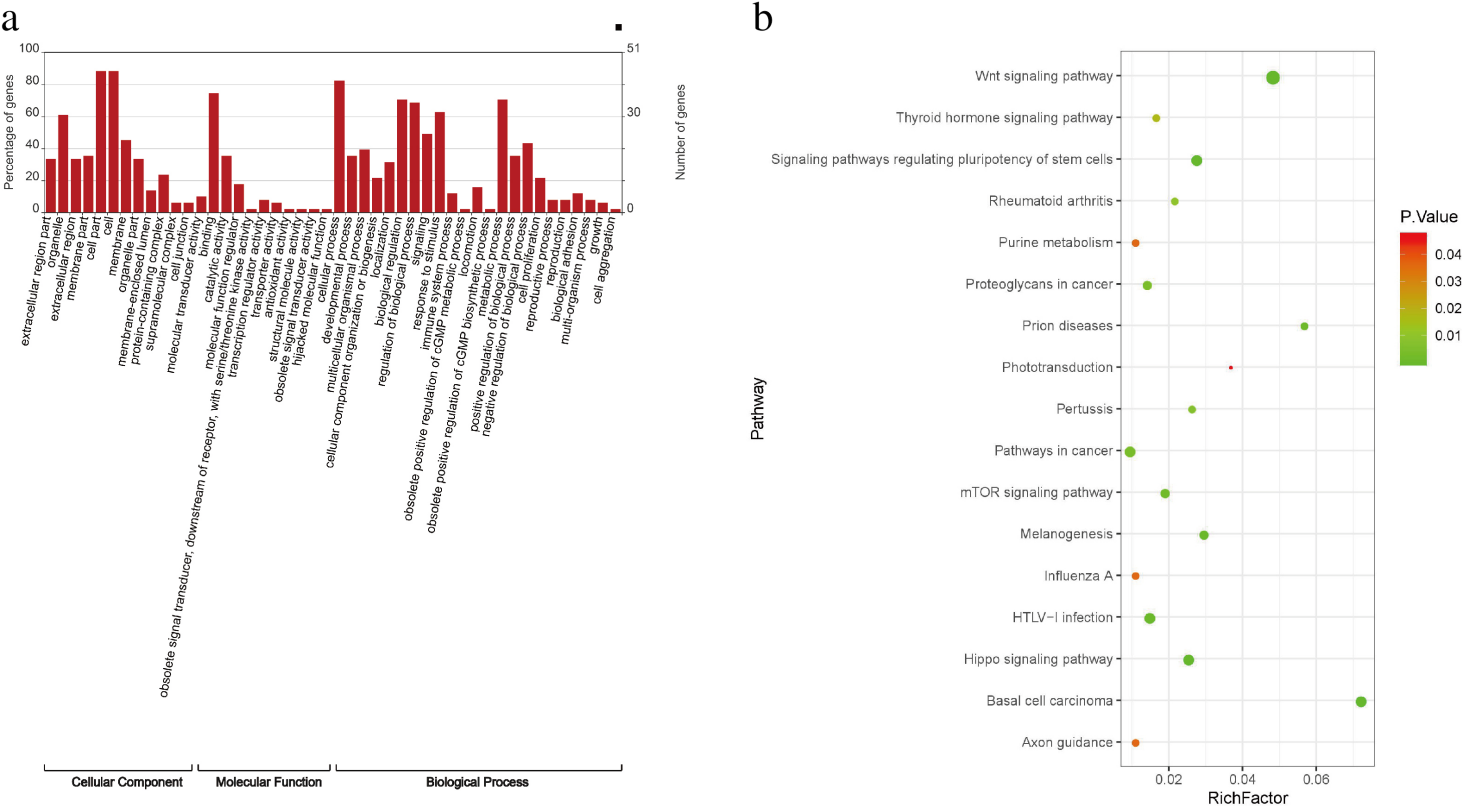
GO and KEGG pathway enrichment analyses for DEGs. (**a**) Graph of GO enrichment analysis for DEGs. (**b**) Bubble plot of KEGG pathway enrichment analysis of DEGs () 1, () 2, () 3, () 4, (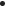) 5, (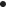) 6, (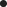) 7. DEG, differentially expressed gene; GO, Gene Ontology; KEGG, Kyoto Encyclopedia of Genes and Genomes.

To investigate interactions between miRNA and mRNA with circ‐IGF1R, we used RNA‐seq for lung cancer PC9 and A549 cell lines overexpressing circ‐IGF1R, and their corresponding normal cell lines (Fig 5a,b). The results showed that there were 68 differentially expressed genes in the PC9 and A549 cells overexpressing circ‐IGF1R (Fig 5c). Next, we carried out GO and KEGG enrichment analyses of the 68 DEGs. This showed that the DEGs were primarily associated with cell components such as the organelles that are involved in molecular functions such as binding and catalytic activities, and biological processes, such as the regulation of the noncanonical Wnt signaling pathway, the regulation of the Wnt signaling pathway, the planar cell polarity pathway, giogenesis, the negative regulation of cell migration and regulation of the JAK‐STAT cascade (Fig 6a). The KEGG pathway analysis also showed that the Wnt signaling pathway and the pathways in cancer were significantly enriched (Fig 6b).

**Figure 7:**
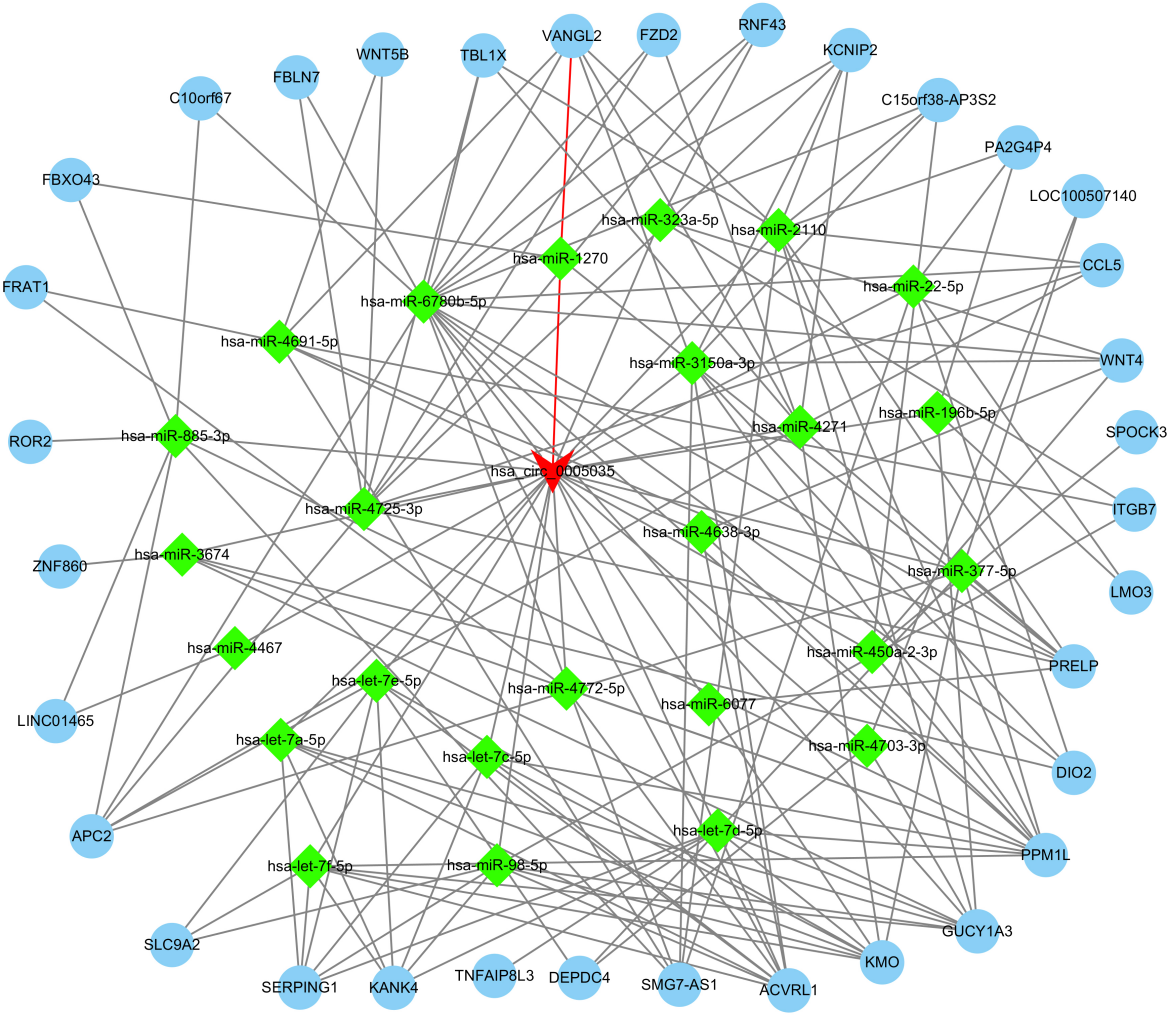
The circ‐IGF1R–miRNA–mRNA network was analyzed by bioinformatics.

**Figure 8:**
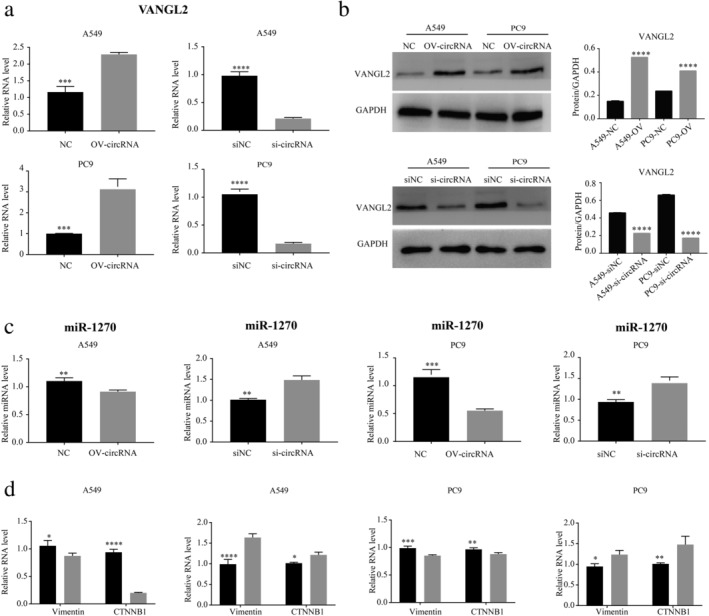
The expression level of VANGL2, miR‐127, CTNNB1, and vimentin in A549 and PC9 lung cancer cell lines was verified using qRT‐PCR and Western Blotting. The expression level of VANGL2 (**a**) is consistent with the overexpression and interference with circ‐IGF1R in A549 and PC9 lung cancer cell lines using qRT‐PCR (**a**) and western blotting (**b**). The expression level of miR‐1270 (**c**) is opposite to the overexpression and interference of circ‐IGF1R in A549 and PC9 lung cancer cell lines. The expression of CTNNB1 and vimentin was downregulated after overexpressing circ‐IGF1R in cells, while the expression of related proteins was upregulated after interference with circ‐IGF1R expression (**d**) (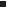) NC, and (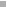) OV‐circRNA; (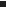) NC, and (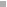) si‐circRNA; (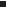) NC, and (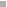) OV‐circRNA; (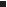) NC, and (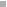) si‐circRNA.

We realized that the target gene of circ‐IGF1R is likely to be involved in the invasion and migration of lung cancer through the Wnt signaling pathway. Through network diagram analysis (Fig 7), qRT‐PCR and western blotting experiments, we finally screened the target gene associated with the Wnt signal pathway (VANGL2). Results obtained using qRT‐PCR (Fig 8a) and western blotting (Fig 8b) showed that the expression of VANGL2 was consistent with that of circ‐IGF1R in the overexpression and interference cell lines, which indicated that the gene may be regulated by circ‐IGF1R.

### Prediction of miRNAs acting on circ‐IGF1R and VANGL2 by bioinformatics

**Figure 9:**
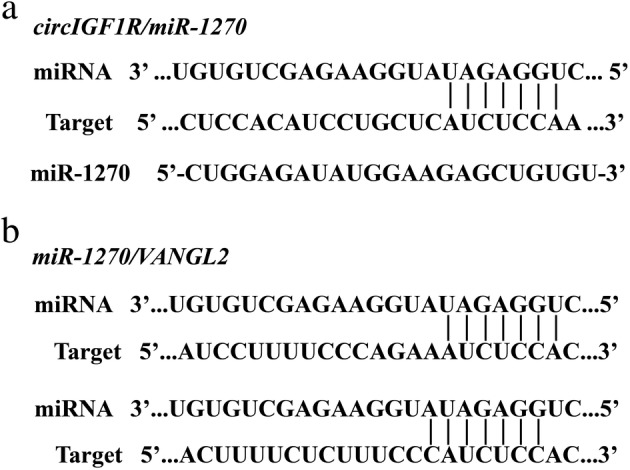
The binding sites of circ‐IGF1R and miR‐1270 and VANGL2 were predicted using bioinformatics.

Studies have shown that circRNA can act as an miRNA sponge and affect the expression of target genes.^5^, ^9^, ^10^, ^11^ We used TargetScan (http://www.targetscan.org/) and miRanda (http://www.microrna.org/microrna/home.do) for predicting miRNA/VANGL2–binding sites; and Circinteractome (https://circinteractome.nia.nih.gov/) for predicting circ‐IGF1R and miRNA‐binding sites. We also verified candidate miRNAs using qRT‐PCR, and successfully screened for the miRNA, miR‐1270 (Fig 9). In the overexpression and interference of the circ‐IGF1R cell line, the results of qRT‐PCR showed that the expression of miR‐1270 was negatively correlated with circRNA (Fig 8c). This observation suggested that miR‐1270 may be regulated by circ‐IGF1R. Thus far, we have identified the potential mechanism of action of circ‐IGF1R/miR‐1270/VANGL2.

### Circ‐IGF1R may affect downstream proteins associated with the Wnt signaling pathway

For further investigating the downstream mechanism of action of the Wnt signaling pathway, we performed qRT‐PCR assays on β‐Catenin1 (CTNNB1) and vimentin. The expressions of the CTNNB1 and vimentin proteins, which are associated with the Wnt pathway, migration and invasion, were assessed. These proteins were downregulated after overexpressing circ‐IGF1R in lung cancer cells, while the expressions of related proteins were upregulated after interference with circ‐IGF1R expression using siRNA (Fig 8d).

## Discussion

At present, research on the clinical diagnosis and treatment of lung cancer, combined with the treatment strategies of immunotherapy, targeted therapy, and chemotherapy, is becoming a new focus in research.^12^ There is an urgent requirement to identify novel molecular markers and therapeutic targets.

CircRNA is characterized by abundant expression and conservation, stable expression in saliva, blood, and exosomes, and CircRNA exhibits specificity at the stage of tissue development.^13^, ^14^, ^15^ In addition, circRNA is more easily detectable than other miRNAs, which means that it can be useful as a tumor biomarker.^16^ Various circRNAs are presently considered potential biomarkers. CircLARP4 inhibits the biological function of gastric cancer (GC) cells by adhering to miR‐424 in GC, and it can influence the overall survival time of patients with GC independent of other factors.^17^

CircMTO1 reduces the carcinogenic effects of miR‐9 and inhibits the progression of hepatocellular carcinoma (HCC), suggesting the potential value of circMTO1 as a therapeutic target for HCC.^18^ Fu *et al*. found that circ‐IGF1R is upregulated in HCC tissues, cell proliferation was significantly attenuated by siRNA knockdown of circ‐IGF1R; circ‐IGF1R may, thus, play a carcinogenic role in HCC by activating the PI3K/AKT signaling pathway.^19^ In addition, Jiang *et al*. performed RNA‐seq on the human bronchial epithelial cell line, BEAS‐2B (BEAS‐2B‐T), transformed by benzo(a)pyrene (BaP), and found that circ‐IGF1R is one of the circRNAs that play a potential role in the transformation of lung cancer cells.^20^ However, the mechanism of action of circ‐IGF1R and diagnosis and treatment using circ‐IGF1R in lung cancer has not yet been clarified.

The present study found that circ‐IGF1R is downregulated in lung cancer, and its expression level is associated with larger tumors and lymph node metastasis, suggesting that circ‐IGF1R may indicate the poor prognosis of patients with lung cancer.

Tumor invasion and migration are important indications for tumor progression because they are able to obtain information on the degree of malignancy and patient prognosis.^21^ Tumor metastasis is a multistage process, which is regulated by several genes, including metastasis‐associated genes and metastasis‐suppressing genes.^22^ A variety of circRNAs affect lung cancer invasion and migration by regulating related targets, such as hsa_circ_0007385, circ‐BANP, and circPTK2.^23^, ^24^, ^25^ In our study, the overexpression of circ‐IGF1R in lung cancer cells inhibited cell migration and invasion and interfered with the ability of circ‐IGF1R to promote cell invasion and migration. However, IGF1R displays potent antiapoptotic, prosurvival capacities and plays a key role in malignant transformation.^26^ We speculate that when IGF1R is transcribed to form pre‐mRNA, linear RNA is upregulated due to a competitive splicing mechanism. When the total amount of pre‐mRNA is unchanged, the corresponding circ‐IGF1R is downregulated. circ‐IGF1R may therefore function in gene regulation by competing with linear splicing.^7^

VANGL2 was discovered in 1998 owing to the important role it plays in planar cell polarity (PCP).^27^ Together with VANGL1, VANGL2 constitutes a PCP scaffold protein. Dysfunction in PCP can lead to the abnormal activation of the developmental pathway and ultimately promotes tumor progression.^28^ Previous study have found that VANGL2 was upregulated in transcripts of ovarian and uterine cancer, and high expression levels are associated with poor prognosis in patients with breast cancer and GBM.^29^, ^30^, ^31^ In contrast, we found that the overexpression of circ‐IGF1R in lung cancer (specifically A549 and PC9 cell lines) resulted in the consistent upregulation of VANGL2 expression, which inhibited the invasion and migration of lung cancer cells. To further investigate the mechanism of action of VANGL2, we found evidence suggesting that VANGL2 can inhibit the canonical Wnt/β‐catenin pathway through a non‐canonical Wnt/PCP signaling pathway.^32^, ^33^ We examined the invasion‐ and migration‐associated proteins, CTNNB1 and vimentin, of the canonical Wnt/β‐catenin pathway using qRT‐PCR. The expression level of VANGL2 increased due to the overexpression of circ‐IGF1R, and the expression levels of CTNNB1 and vimentin were reduced, which revealed that an increase in VANGL2 expression may inhibit the Wnt/β‐catenin pathway in lung cancer cells, which in turn affects cancer cell invasion and migration.

CircRNA can act as an miRNA sponge to regulate its function and influence gene expression. Numerous studies have shown that the circRNA–miRNA–mRNA axis plays a key role in cancer‐associated pathways.^34^, ^35^, ^36^ By performing a binding site analysis of circ‐IGF1R–miRNA–VANGL2 and qRT‐PCR validation, we finally selected hsa‐miR‐1270. Previous studies have found that miR‐1270 is upregulated in osteosarcoma, human renal cancer, and papillary thyroid cancer.^37^, ^38^, ^39^ The lentivirus‐induced downregulation of miR‐1270 inhibits cancer proliferation and migration. Our study also showed that the overexpression of circ‐IGF1R in lung cancer resulted in the downregulation of miR‐1270 expression and the inhibition of cell invasion and migration. In contrast, in glioblastoma multiforme,^40^ miR‐1270 expression is downregulated, suggesting tumor heterogeneity in different tumors.

To the best of our knowledge, we have identified for the first time that circ‐IGF1R can inhibit the Wnt/β‐catenin pathway through the circ‐IGF1R–miR‐1270–VANGL2 axis in lung cancer, thereby inhibiting cell invasion and migration. Previous studies have found that extracellular matrix degradation, tumor angiogenesis, cell adhesion, and the tumor microenvironment affect lung cancer invasion and migration to different degrees.^41^, ^42^, ^43^, ^44^ We analyzed the transcriptional profile of lung cancer cell lines expressing circ‐IGF1R using bioinformatics. The results suggested that circ‐IGF1R plays a part in the invasion and migration of lung cancer by regulating important miRNAs and genes. Possible biological processes include the regulation of the non‐canonical Wnt signaling pathway, giogenesis, the PCP pathway, the negative regulation of cell migration, and the regulation of the JAK‐STAT cascade. Interestingly, we found that the mTOR signaling pathway downstream of PI3K/AKT is also enriched. The mTOR signaling is mainly activated through the PI3K‐AKT‐mTOR pathway, and was found to be associated with high grade tumors (G3‐G4) and advanced disease (stage III) of lung cancer.^45^ Accordingly inhibitors of PI3K signaling have been suggested as potential therapeutic agents in NSCLC.^46^, ^47^ Some studies have found that PI3K‐AKT‐mTOR may negatively regulate the classic WNT signaling pathway through the downstream effector GSK3β.^48^, ^49^ The relationship between circ‐IGF1R and key factors such as GSK3β may also be worth exploring in the future. It was revealed that circ‐IGF1R not only affects HCC through PI3K‐AKT‐mTOR, but also may promote the growth and metastasis of lung cancer through PI3K‐AKT‐mTOR. These BPs support our research perspective and are consistent with previous research findings.

In conclusion, we have shown that the low expression level of circ‐IGF1R is detected in lung cancer tissues and cell lines. Circ‐IGF1R may inhibit lung cancer invasion and migration through a network of circ‐IGF1R–miR‐1270–VANGL2.

## Disclosure

The authors declare that there are no conflicts of interest.
